# Novel *ACTG1* mutations in patients identified by massively parallel DNA sequencing cause progressive hearing loss

**DOI:** 10.1038/s41598-020-63690-5

**Published:** 2020-04-27

**Authors:** Hiroki Miyajima, Hideaki Moteki, Timothy Day, Shin-ya Nishio, Takaaki Murata, Tetsuo Ikezono, Hidehiko Takeda, Satoko Abe, Satoshi Iwasaki, Masahiro Takahashi, Yasushi Naito, Hiroshi Yamazaki, Yukihiko Kanda, Shin-ichiro Kitajiri, Shin-ichi Usami

**Affiliations:** 1 Department of Otorhinolaryngology, Shinshu University School of Medicine, Matsumoto, Japan; 2 Department of Hearing Implant Sciences, Shinshu University School of Medicine, Matsumoto, Japan; 3 Murata Otorhinolaryngology Clinic, Numata, Japan; 4 Department of Otorhinolaryngology, Saitama Medical University, Irima, Japan; 5 Department of Otorhinolaryngology, Toranomon Hospital, Tokyo, Japan; 6 Department of Otorhinolaryngology, International University of Health and Welfare, Mita Hospital, Tokyo, Japan; 7 Departments of Otolaryngology - Head and Neck Surgery, Kobe City Medical Center General Hospital, Kobe, Japan; 8 Department of Otolaryngology, Osaka Red Cross Hospital, Osaka, Japan; 9 Kanda ENT Clinic, Nagasaki Bell Hearing Center, Nagasaki, Japan

**Keywords:** Clinical genetics, Disease genetics

## Abstract

Human *ACTG1* mutations are associated with high-frequency hearing loss, and patients with mutations in this gene are good candidates for electric acoustic stimulation. To better understand the genetic etiology of hearing loss cases, massively parallel DNA sequencing was performed on 7,048 unrelated Japanese hearing loss probands. Among 1,336 autosomal dominant hearing loss patients, we identified 15 probands (1.1%) with 13 potentially pathogenic *ACTG1* variants. Six variants were novel and seven were previously reported. We collected and analyzed the detailed clinical features of these patients. The average progression rate of hearing deterioration in pure-tone average for four frequencies was 1.7 dB/year from 0 to 50 years age, and all individuals over 60 years of age had severe hearing loss. To better understand the underlying disease-causing mechanism, intracellular localization of wild-type and mutant gamma-actins were examined using the NIH/3T3 fibroblast cell line. *ACTG1* mutants p.I34M p.M82I, p.K118M and p.I165V formed small aggregates while p.R37H, p.G48R, p.E241K and p.H275Y mutant gamma-actins were distributed in a similar manner to the WT. From these results, we believe that some part of the pathogenesis of *ACTG1* mutations may be driven by the inability of defective gamma-actin to be polymerized into F-actin.

## Introduction

Autosomal dominant non-syndromic hearing loss (ADNSHL) occurs in about 20% of non-syndromic hereditary hearing loss (HL) cases^1^, and 38 genes have been reported to be associated with ADNSHL (Van Camp G, Smith RJH. Hereditary Hearing Loss Homepage: http://hereditaryhearingloss.org).

The emergence of massively parallel DNA sequencing has allowed the rapid and cost-effective detection of disease-causing variants, and it is already available for particularly effective medical care based on the accurate diagnosis of Mendelian disorders^2^.

Among the 38 genes associated with ADNSHL, *ACTG1*(OMIM: *102560) has received special attention for several reasons. First, *ACTG1-*associated HL (DFNA20/26, OMIM: #604717) patients show high-frequency progressive HL^3^ and are good candidates for electric acoustic stimulation (EAS)^4^. Second, the *ACTG1*-encoding protein, γ (gamma)-actin, is a component of the well-studied stereocilia^5–9^, and the functional consequences of some *ACTG1* mutations can be analyzed by molecular biology^5^. Hair cell stereocilia are crucial for converting the mechanical forces of sound waves into electrical signals (i.e., mechanotransduction)^10^. These specialized structures are located on the apical surface of auditory hair cells, and are highly dependent on their actin cytoskeletons^11^.

To date, 33 cases with *ACTG1-*associated non-syndromic HL have been reported. However, the detailed clinical features, including the rate of progression of HL, remain unclear. Therefore, we sought to clarify the clinical features of HL patients with *ACTG1* mutations by the genomic sequencing of a large cohort and the functional analysis of *ACTG1* mutations to gain a further understanding of the underlying disease-causing mechanism.

## Materials and Methods

### Study subjects

All procedures were approved by the Shinshu University Ethical Committee as well as the respective Ethical Committees of the other participating institutions described below. Akita University Ethical Committee, Iwate Medical University Ethical Committee, Tohoku Rosai Hospital Ethical Committee, Fukushima Medical University Ethical Committee, Yamagata University Ethical Committee, Dokkyo Medical University Ethical Committee, TAKASAKI Ear Nose & Throat Clinic Ethical Committee, Niigata University Ethical Committee, Tokyo Medical University Ethical Committee, Jikei University Ethical Committee, Toranomon Hospital Ethical Committee, Kitasato University Ethical Committee, International University of Health and Welfare Mita Hospital Ethical Committee, National Rehabilitation Center for Persons with Disabilities Ethical Committee, Keio University Ethical Committee, Hamamatsu University Ethical Committee, Shiga University Ethical Committee, Shiga Medical Center for Children Ethical Committee, Osaka University Ethical Committee, Kobe City Medical Center General Hospital Ethical Committee, Hyogo College of Medicine Ethical Committee, Kyoto Prefectural University Ethical Committee, Okayama University Ethical Committee, Yamaguchi University Ethical Committee, Ehime University Ethical Committee, Kyushu University Ethical Committee, Kanda ENT Clinic Ethical Committee, Nagasaki University Ethical Committee, Miyazaki University Ethical Committee, Kagoshima University Ethical Committee, Ryukyus University Ethical Committee, Sapporo Medical University Ethical Committee, Tohoku University Ethical Committee, Jichi Medical University Ethical Committee, Gunma University Ethical Committee, Jyuntendo University Ethical Committee, Yokohama City University Ethical Committee, Mejiro University Ethical Committee, Saitama Medical University Ethical Committee, Abe ENT clinic Ethical Committee, Tokyo Medical Center Institute of Sensory Organs Ethical Committee, Jichi University Saitama Medical Center Ethical Committee, Aichi Children’s Health Medical Center Ethical Committee, Chubu Rosai Hospital Ethical Committee, Kyoto University Ethical Committee, Mie University Ethical Committee, Kansai Medical University Ethical Committee, Kobe University Ethical Committee, Osaka Medical Center and Research Institute for Maternal and Children Health Ethical Committee, Wakayama Medical University Ethical Committee, Kouchi University Ethical Committee, Hiroshima University Ethical Committee, Hiroshima City Hiroshima Citizen Hospital Ethical Committee, Fukuoka University Ethical Committee, Kurume University Ethical Committee, National Defense Medical College Ethical Committee, Tokai University Ethical Committee, Hokkaido University Ethical Committee, Kanagawa Children’s Medical Center Ethical Committee, Tokyo Medical and Dental University Ethical Committee, Hirosaki University Ethical Committee, Tokyo Metropolitan Children’s Medical Center Ethical Committee, Hakodate Central General Hospital Ethical Committee, Osaka Red Cross Hospital Ethical Committee, Hiroshima Prefectural Hospital Ethical Committee, Nara Medical University Ethical Committee, and Tsukuba University Ethical Committee. All methods were performed in accordance with the Guidelines for Genetic Tests and Diagnoses in Medical Practice of the Japanese Association of Medical Sciences and the Declaration of Helsinki as required by Shinshu University. Written informed consent was obtained from all subjects (or from their next of kin, caretaker, or guardian in the case of minors/children). A total of 7,408 probands from unrelated Japanese hearing loss families were enrolled between February 2012 and October 2017 from the 67 otolaryngology departments participating in the present study from across Japan. The hereditary patterns of the hearing loss in the probands’ families were autosomal dominant in 1,336 cases, autosomal recessive or sporadic in 5,564 cases, and unknown inheritance pattern in 1,174 cases. Among the 7,408 probands, 1,120 probands (266 autosomal dominant, 600 autosomal recessive or sporadic and 254 unknown family history) overlapped with our previous study cohort^4^.

### Methods

#### Amplicon resequencing with MPS

Amplicon libraries were prepared using an Ion AmpliSeq Custom Panel (Applied Biosystems, Life Technologies), in accordance with the manufacturer’s instructions, for 68 genes reported to cause non-syndromic hereditary HL (Supplementary Table 1). The detailed sample preparation protocol has been described elsewhere^12,13^. Sequencing was performed in accordance with the manufacturer’s instructions. Massively Parallel Sequencing (MPS) was performed with an Ion Torrent Personal Genome Machine (PGM) system or Ion Proton System using an Ion PGM 200 Sequencing Kit with an Ion 318 Chip (Life Technologies) or Ion HiQ Chef Kit with an Ion P1 chip. The sequence data were mapped against the human genome sequence (build GRCh37/ hg19) with a Torrent Mapping Alignment Program. After sequence mapping, the DNA variants were detected with Torrent Variant Caller plug-in software. After variant detection, their effects were analyzed using ANNOVAR software^14^.

### Variant prioritization and pathogenicity classification

In previous reports, the inheritance pattern for *ACTG1-*associated hearing loss was shown to be autosomal dominant^8^. Thus, we selected hearing loss patients from apparent autosomal dominant families, selecting the patients with *ACTG1* variants from among 1,336 autosomal dominant HL families including all autosomal dominant HL families in our previous reports^4,12^. The missense, nonsense, insertion/deletion and splicing variants were selected from among the identified variants for further analysis. Variants were further selected as less than 1% of 1) the 1,000 genome database^15^, 2) the 6,500 exome variants^16^, 3) the Human Genetic Variation Database (dataset for 1,208 Japanese exome variants)^17^, and 4) the 333 in-house Japanese normal hearing controls. Direct sequencing was utilized to confirm the selected variants.

The pathogenicity of the identified variants was evaluated according to the American College of Medical Genetics (ACMG) standards and guidelines^18^. Based on the ACMG guidelines, we regarded “pathogenic” and “likely pathogenic” variants as strong candidates for *ACTG1-*associated hearing loss. In addition, we listed the “variants of uncertain significance” remaining after the filtering procedure. Among the novel “variants of uncertain significance”, we removed those with Combined Annotation Dependent Depletion (CADD) Phred scores of less than 15 as these variants appear to be non-pathogenic and not depleted from the general control population. Family segregation analysis was performed for each proband and their family members by Sanger sequencing. We refer to the minor allele frequencies for the identified *ACTG1* variants based on the Exome Aggregation Consortium (ExAC) database (http://exac.broadinstitute.org) and gnomAD database (https://gnomad.broadinstitute.org).

### Clinical evaluations

The age at onset of HL, the progressiveness of HL and history of vertigo/dizziness were analyzed based on medical charts. Pure-tone audiometry was performed to evaluate hearing thresholds. Conditioned orientation response (COR) audiometry^19^ was performed in pediatric cases instead of pure-tone audiometry. Pure-tone average (PTA) was calculated from the audiometric thresholds at four frequencies (0.5, 1, 2, and 4 kHz). If an individual did not respond to the maximum hearing level at a frequency, 5 dB was added to the maximum hearing level. The severity of HL was classified into mild (PTA: 21–40 dB HL), moderate (41–70 dB HL), severe (71–95 dB HL), and profound (>95 dB HL). Intervention for HL, including the use of hearing aids, cochlear implants and EAS, was investigated based on medical charts.

### *In vitro* analysis

#### Mutagenesis

The novel mutations c.102 C > G, c.110 G > A, c.246 G > A, c.493 C > G c.823 C > T, and c.994 C > T, and the previously reported variants c.142 G > C, c.266 C > T, c.353 A > T, c.354 G > C, c.721 G > A, c.791 C > T, c.895 C > G and c.914 T > C were introduced into the *ACTG1* plasmid (pFN21A HaloTag CMV *ACTG1* plasmid: purchased from Kazusa DNA Research Lab, FHC07847) according to manufacturer’s protocol using an In-Fusion HD Cloning Plus Kit (Takara Bio, Shiga, Japan). All variants mentioned in this study were shown in NM_001614.

Briefly, PCR was performed with the mutation-specific primers under the following conditions: 35 cycles of 98 °C for 10 sec, 58 °C for 30 sec, and 72 °C for 5 min. After the PCR reaction, 5μl of the PCR products was digested with *Dpn*I at 37 °C for 5 hours to remove the template plasmid, and then deactivated at 80 °C for 15 minutes. Next, recombination of the PCR products was induced with infusion recombinase.

After recombination, plasmids were transformed into Top10 competent cells (Thermo Fisher, MA, USA) for amplification. Sanger sequencing was used to confirm all variants.

### Cell culture

Morin *et al*. reported that *ACTG1* mutant constructs expressed in NIH/3T3 fibroblasts formed aggregates^5^. We used the same cell line to evaluate the effects of actin mutations and allow comparison with the results of their report.

NIH/3T3 cells were cultured in DMEM supplemented with 10% fetal calf serum (FCS) on 15-mm glass coverslips (Matsunami Glass, Osaka, Japan) in flat-bottomed 12-well multititer plates (Iwaki Glass, Shizuoka, Japan) for immunocytochemical study.

### Transfection with wild-type and mutant vectors

NIH/3T3 cells were transfected with 1μg of the wild-type γ-actin plasmid or each of the 11 mutant plasmids using Lipofectamine 3000 (Thermo Fisher, MA, USA) according to the manufacturer’s instructions.

### Immunocytochemistry

For immunocytochemistry, cells on coverslips were fixed in 4% paraformaldehyde-phosphate buffer for 10 min at 48 h after transfection, and then washed three times briefly in PBS. To study intracellular protein localization, cells were permeabilized for 20 min at room temperature with 0.1% Triton X-100 (SIGMA, Kanagawa, Japan) in PBS, blocked with 3% Goat serum (Gibco, Thermo Fisher, MA, USA) in PBS for 20 min, and incubated for 1 h at room temperature with a 1:200 dilution of AntiHalo-Tag pAb (Promega, Tokyo, Japan). Cells were washed three times with PBS, and then incubated for 1 h at room temperature with a 1:200 dilution of the secondary antibody, Alexa Fluor 546 goat anti-rabbit antibody. Total actin was stained with Alexa Fluor 488 phalloidin (Invitrogen, Thermo Fisher, MA, USA), and a 1/1000 dilution of DAPI (KPL, MA, USA) to visualize the nucleus. Cells were washed in PBS 3 times, and coverslips were mounted onto a slide glass with Pro-long Gold Antifade Mountant (Invitrogen, Thermo Fisher, MA, USA). Images were taken with an Olympus Fluoview FV-10i system (Olympus, Okaya, Japan).

## Results

### *ACTG1* gene variants identified in this study

**Figure 1:**
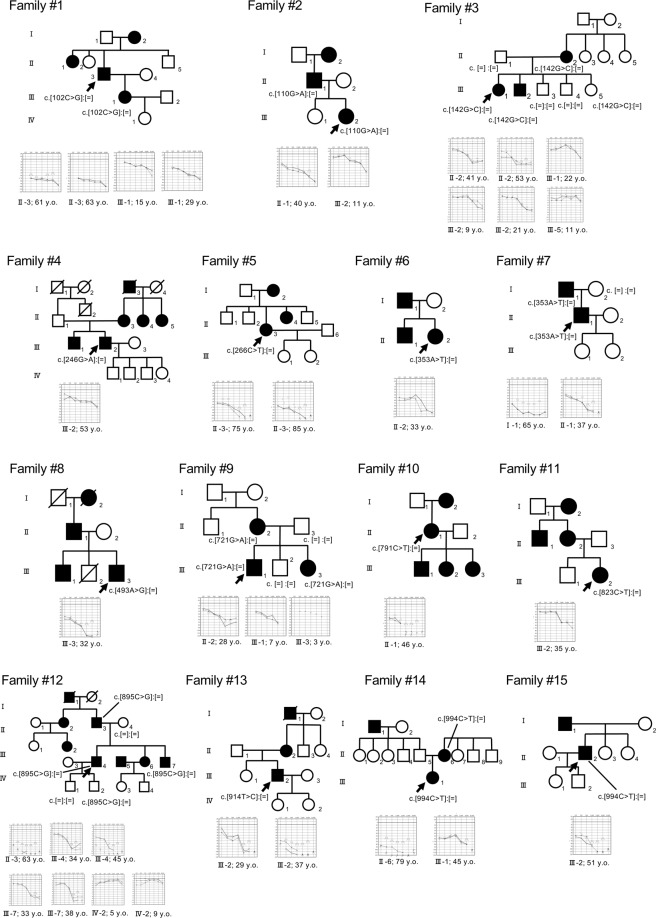
Pedigree and audiograms for each family with an *ACTG1* variant. Arrows show the probands in each family. Genetic findings for each individual are shown in the pedigree.

The MPS screening of the 1,336 ADNSHL patients identified 13 possibly pathogenic variants in the *ACTG1* gene from 15 unrelated families, including four mutations from five families in our previous reports^4^ (Family 3, 6, 7, 9, and 12) (Fig. 1). Among the 13 variants, six were novel (c.102 C > G, c.110 G > A, c.246 G > A, c.493 C > G c.823 C > T, and c.994 C > T) and seven were previously reported variants (c.142 G > C, c.266 C > T, c.353 A > T, c.721 G > A, c.791 C > T, c.895 C > G and c.914 T > C). None of the candidate *ACTG1* variants were observed in the ExAC database. In addition, 12 of the 13 variants identified in this study were not observed in the gnomAD database. Among the 13 identified variants, 4 were classified as “pathogenic” variants and 3 were classified as “likely pathogenic” variants; however, 6 remained as “variants of uncertain significance” based on the ACMG guidelines^18^ (Table 1).

**Table 1:** Possible causative *ACTG1* variants in this study.

Nucleotide	Amino acid	Allele frequency	CADD Phred score	ACMG criteria	Supporting evidence	Reference	
change	change	(gnomAD browser)					
c.102 C > G	p.I34M	3.98E-06	21.5	Uncertain Significance	PM2	This study	
c.110 G > A	p.R37H	0	16.11	Uncertain Significance	PM2 + PP1	This study	
c.142 G > C	p.G48R	0	26.6	Uncertain Significance	PM2 + PP1 + PP3	Miyagawa *et al*., 2015	
c.246 G > A	p.M82I	0	24.5	Uncertain Significance	PM2 + PP3	This study	
c.266 C > T	p.T89I	0	19.88	Pathogenic	PS4_Moderate+PM2 + PP1_Strong+PP3	Zhu *et al*., 2003	
c.353 A > T	p.K118M	0	26.1	Pathogenic	PS4_Moderate+PM2 + PP1_Strong+PP3	Zhu *et al*., 2003	Miyagawa *et al*., 2015
c.493 A > G	p.I165V	0	20.7	Uncertain Significance	PM2 + PP1 + PP3	This study	
c.721 G > A	p.E241K	0	24.7	Pathogenic	PS4_Moderate+PM2 + PP1_Strong+PP3	Morin *et al*., 2009	Miyagawa *et al*., 2015
c.791 C > T	p.P264L	0	24.2	Pathogenic	PS4_Moderate+PM2 + PP1_Strong+PP3	Zhu *et al*., 2003	
c.823 C > T	p.H275Y	0	23.8	Uncertain Significance	PM2 + PP1	This study	
c.895 C > G	p.L299V	0	19.96	Likely Pathogenic	PS4_Supporting+PM2 + PP1_Strong+PP3	Miyagawa *et al*., 2015	
c.914 T > C	p.M305T	0	24.2	Likely Pathogenic	PS4_Moderate+PM2 + PP1_Strong+PP3	Park *et al*., 2013	
c.994 C > T	p.P332S	0	10.27	Likely Pathogenic	PS4_Supporting+PM2 + PM5 + PP1	This study	

Abbreviations in the Supporting evidence row are: PS, Strong evidence of pathogenicity; PM, Moderate evidence of pathogenicity; PP, Supporting evidence of pathogenicity.

### Clinical characteristics of *ACTG1*-associated HL patients

Table 2 summarizes the clinical characteristics of 27 affected individuals from 15 families with the candidate *ACTG1* variants. The age at onset of HL varied markedly from 3 to 59 years. Among the 27 affected individuals, 3 cases showed mild HL, 11 cases moderate HL, 5 cases severe HL and 4 cases profound HL. It is noteworthy that 4 cases with the candidate *ACTG1* variants had normal hearing. Most of these cases were of a younger age and, based on their family history, type of HL, and age at onset information, we suspected that these cases had HL in only the higher frequencies (for example: Family 2 III-2, Family 3 III-1, and III-2 [Fig. 1]) or would develop HL after several years. Progression of HL was noticed in 23 (85%) of the 27 individuals. Tinnitus and episodes of vertigo and/or dizziness were present in 14 (52%) and 4 (14%) of the 27 individuals, respectively. The vestibular tests (caloric test and cervical vestibular evoked myogenic potential [cVEMP]) findings for 2 patients (Family 12 III-4 and Family 13 III -2 [Fig. 1]) were normal. In terms of intervention for HL, we obtained information for 21 individuals, with 13 of them using hearing aids and 2 individuals receiving EAS surgery.

**Table 2:** Clinical characteristics of 27 affected individuals from 15 families with candidate *ACTG1* variants.

Family	Patient	Exon	Nucleotide	Amino acid	Age (y)	Onset	Gender	Hearing	Progression	Tinnitus	Vertigo	Intervention
No.			change	change		Age (y)		Level (dB)				
1	II-3	2	c.102 C > G	p.I34M	64	18	Male	77.5	Progressive	+	+	Hearing aids
1	III-1	2	c.102 C > G	p.I34M	29	15	Female	52.5	Progressive	+	+	None
2	III-2	2	c.110 G > A	p.R37H	11	Unknown	Female	22.5	Unknown	-	-	None
2	II-1	2	c.110 G > A	p.R37H	40	10	Male	63.75	Progressive	+	+	Unknown
3	II-2	3	c.142 G > C	p.G48R	53	11	Female	56.25	Progressive	-	-	Hearing aids
3	III-1	3	c.142 G > C	p.G48R	22	N/A	Female	16.25	Progressive	Unknown	Unknown	None
3	III-2	3	c.142 G > C	p.G48R	9	7	Male	20	Progressive	+	Unknown	None
3	III-5	3	c.142 G > C	p.G48R	11	Precritical	Female	15	Unknown	Unknown	Unknown	None
4	III-2	3	c.246 G > A	p.M82I	53	Unknown	Male	41.25	Unknown	-	-	None
5	II-3	3	c.266 C > T	p.T89I	85	Unknown	Male	83.75	Progressive	+	-	Hearing aids
6	II-2	3	c.353 A > T	p.K118M	33	26	Female	48.75	Progressive	+	+	Hearing aids
7	I-1	3	c.353 A > T	p.K118M	65	17	Male	105	Progressive	Unknown	Unknown	Hearing aids
7	II-1	3	c.353 A > T	p.K118M	37	17	Male	68.75	Progressive	+	-	Hearing aids
8	III-3	4	c.493 A > G	p.I165V	32	11	Male	93.75	Progressive	-	-	Hearing aids
9	II-2	4	c.721 G > A	p.E241K	28	14	Female	51.25	Progressive	-	-	Hearing aids
9	III-1	4	c.721 G > A	p.E241K	7	3	Male	45	Progressive	-	-	Hearing aids
9	III-3	4	c.721 G > A	p.E241K	3	3	Female	38.75	Unknown	Unknown	Unknown	None
10	II-1	4	c.791 C > T	p.P264L	46	12	Female	101.25	Progressive	+	+	Unknown
11	III-2	5	c.823 C > T	p.H275Y	35	34	Female	30	Progressive	+	-	None
12	II-3	5	c.895 C > G	p.L299V	63	46	Male	107.5	Progressive	+	-	Hearing aids
12	III-4	5	c.895 C > G	p.L299V	41	6	Male	58.75	Progressive	+	-	EAS
12	III-7	5	c.895 C > G	p.L299V	38	15	Male	61.25	Progressive	+	-	Hearing aids
12	IV-2	5	c.895 C > G	p.L299V	9	Precritical	Male	6.25	Progressive	-	-	None
13	III-2	5	c.914 T > C	p.M305T	34	6	Male	72.5	Progressive	+	-	EAS
14	II-6	6	c.994 C > T	p.P332S	79	59	Male	107.5	Progressive	+	-	Hearing aids
14	III-1	6	c.994 C > T	p.P332S	45	38	Male	63.75	Progressive	-	-	Unknown
15	III-2	6	c.994 C > T	p.P332S	51	35	Male	91.25	Progressive	-	-	Hearing aids

Abbreviations: y, year(s) old; N/A, not available; EAS, electric acoustic stimulation.

### Rate of deterioration in hearing loss

**Figure 2:**
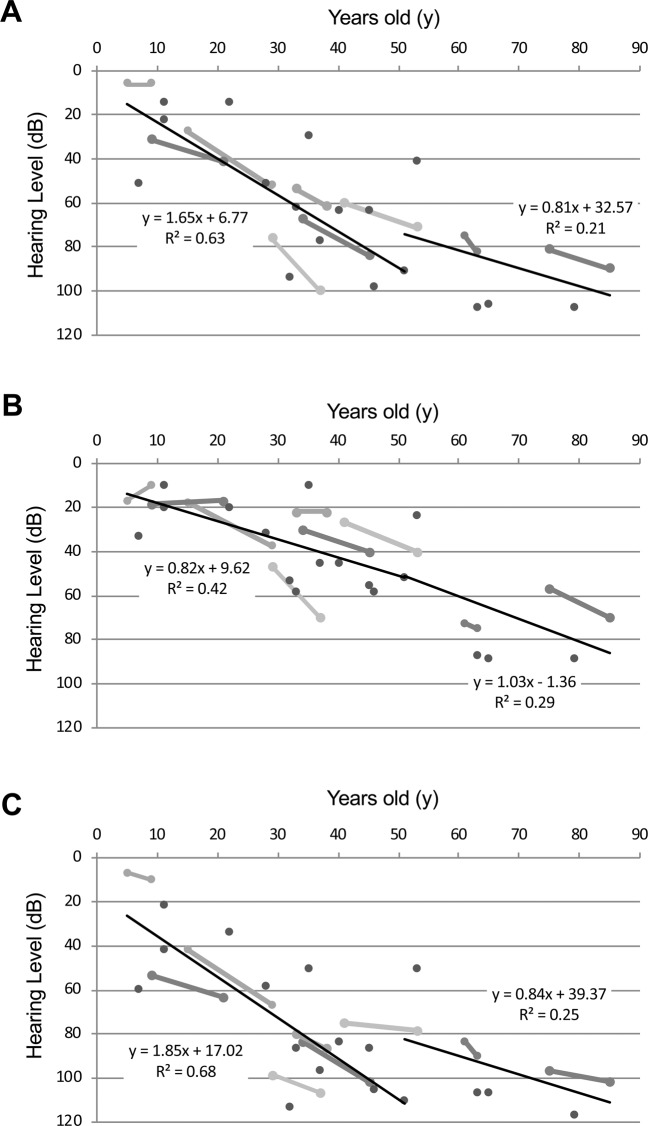
Rate of hearing loss deterioration for lower, middle and higher frequencies. The average progression rates of hearing deterioration in PTA for four frequencies (500, 1000, 2000, 4000 Hz) (**A**), lower frequencies (125,250,500 Hz) (**B**), and higher frequencies (2000,4000,8000 Hz) (**C**). Each solid line indicates the hearing thresholds of the same individual. Thin lines show the average progression rate of hearing deterioration.

**Figure 3:**
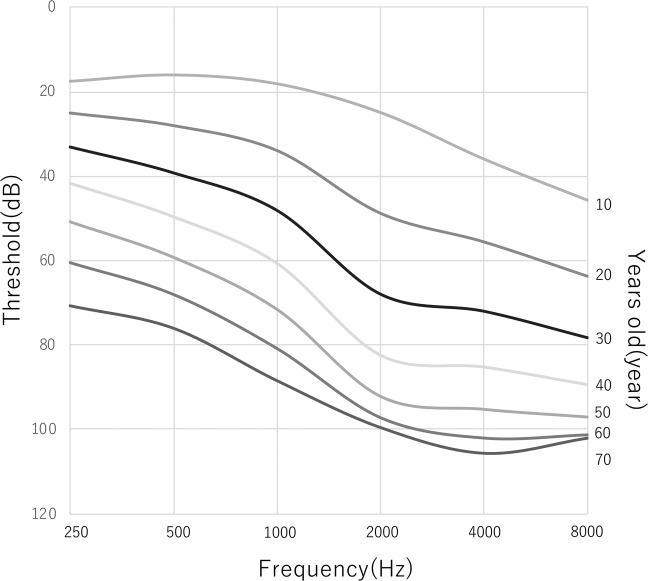
An age-related typical audiogram (ARTA) demonstrating the progression of hearing loss for *ACTG1*.

The rate of deterioration of HL for each frequency was analyzed using the audiogram data for each patient. As shown in Fig. 2, we plotted the high-tone, low-tone and mid-tone averaged hearing thresholds and age. The average progression rate of hearing deterioration in PTA for four frequencies (500, 1000, 2000, 4000 Hz) was 1.7 dB/year at 0 to 50 years age (Fig. 2A). All individuals aged over 60 years had severe HL. Figure 2B,C show the progression rate of hearing deterioration in the lower frequencies (125, 250, 500 Hz), and higher frequencies (2000, 4000, 8000 Hz) for the same individuals respectively. The average progression rates in the lower frequencies were 0.8–1.0 dB/year, whereas they were 1.9 dB/year for the higher frequencies. Thus, the hearing thresholds in the higher frequencies deteriorated more rapidly than did those in the lower frequencies in patients with *ACTG1* variants. Construction of an age-related typical audiogram (ARTA)^20^ showed the same results regarding the rate of HL deterioration (Fig. 3).

### Intracellular localization of WT and mutant γ-actins in NIH/3T3 cells

To understand the putative disease-causing mechanisms, the intracellular localization of wild-type and mutant γ-actins with deafness-associated variants (*ACTG1*:NM_001614: c.102 C > G: p.I34M, c.110 G > A: p.R37H, c.142 G > C: p.G48R, c.246 G > A: p.M82I, c.354 G > C: p.K118N, c.493 C > G: p.I165V, c.721 G > A: p.E241K and c.823 C > T: p.H275Y) was analyzed by expression assay utilizing the NIH/3T3 fibroblast cell line.

**Figure 4:**
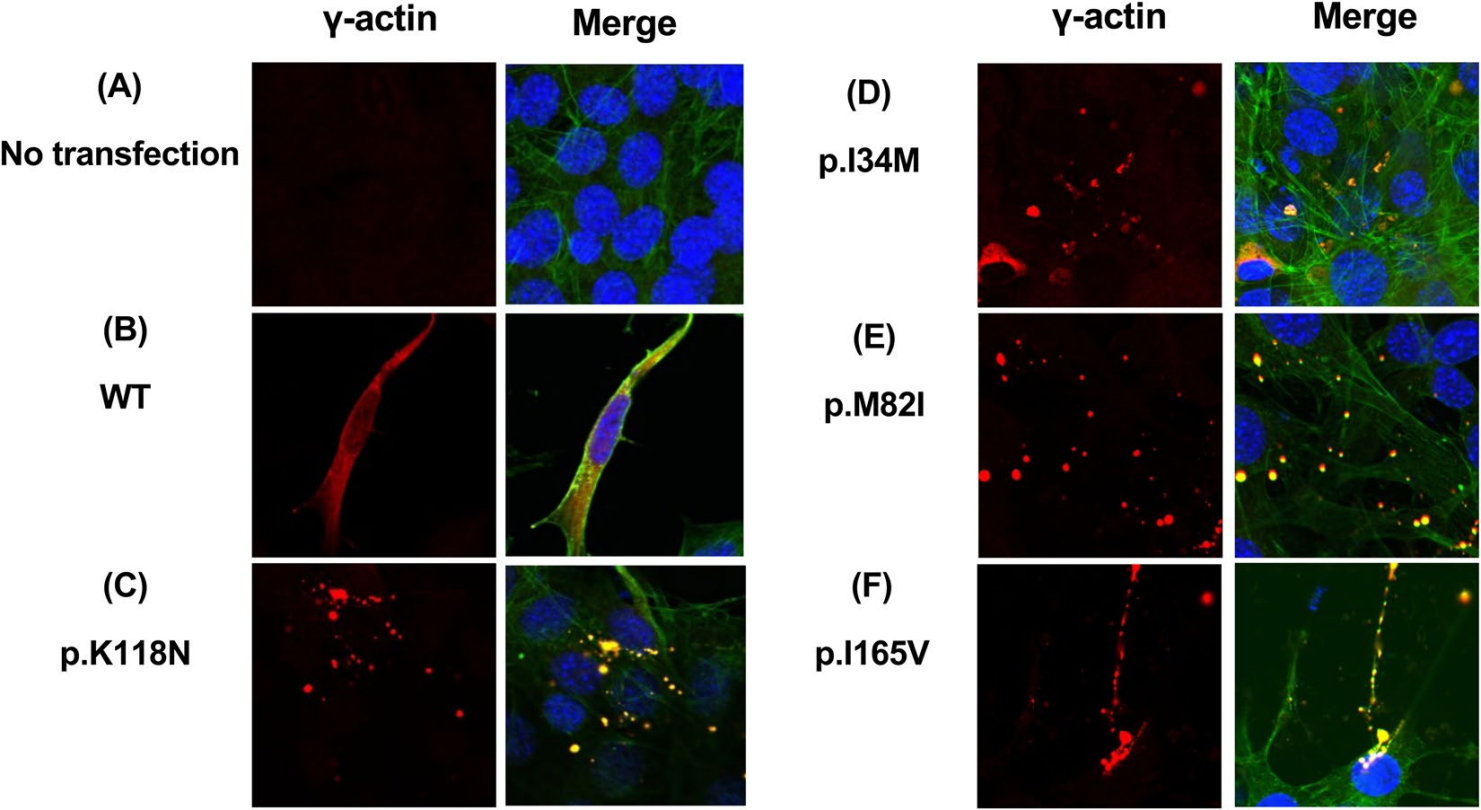
Mutant γ-actin proteins failed to be incorporated into the F-actin network (1). (**A-F**) Confocal images of NIH/3T3 cells transfected with either wild-type or mutant halo-tagged γ-actin (red) analyzed by immunocytochemistry. F-actin localization was detected by phalloidin staining (green) and nuclear staining by DAPI (blue). (**A**) Untransfected cells show low background expression. (**B**) WT γ-actin exhibits smooth incorporation into the F-actin. (**C**) The previously published *ACTG1*:c.354 G > C:p.K118N failed to be incorporated into the F-actin, instead forming aggregates. (**D, E, F**) Mutations identified in this study, *ACTG1*:c.102 C > G:p.I34M, c.246 G > A:p.M82I, c.493 C > G:p.I165V, failed to incorporate into F-actin as marked by phalloidin staining, instead forming aggregates.

In a previous study, a mutant *ACTG1*:NM_001614: c.354 G > C: p.K118N construct expressed in NIH/3T3 fibroblasts did not co-localize with actin stress fibers, but instead formed aggregates^5^. In this study, we also expressed the p.K118N variant as a positive control. Wild-type γ-actin was incorporated into the stress fibers, filamentous actin in ruffles and lamellipodia, and into the actin network, co-localizing with endogenous filamentous actin as visualized with phalloidin (Fig. 4). The p.I34M p.M82I, p.K118M and p.I165V mutant γ-actins formed small aggregates (Fig. 4), while the expression of the p.R37H, p.G48R, p.E241K and p.H275Y mutant γ-actins resembled that of the WT (Supplementary Fig. 1).

## Discussion

Mutations in *ACTG1* represent a rare causative factor for ADNSHL and only a limited number of cases have been reported to date. In this study, MPS facilitated the efficient detection of causative variants for *ACTG1-*associated HL. As a result, six novel variants and seven previously reported variants were successfully identified from 15 unrelated ADNSHL families. The incidence of *ACTG1-*associated HL was 1.1% (15/1336) in the presumably autosomal dominant HL families. This finding shows that *ACTG1* mutations are the fifth most frequent cause of autosomal dominant HL in the Japanese population, after *KCNQ4* (6.6%)^21^, *TECTA* (2.9%)^22^, *POU4F3* (2.7%)^23^, and *WFS1* (2.5%)^24^.

In general, *ACTG1-*associated HL affects the higher frequencies initially and progresses to all frequencies later^7^. Indeed, our results also supported this trend, with HL observed to deteriorate more rapidly in the higher frequencies than in the lower frequencies. However, the onset and severity of HL varied among the patients. Morin *et al*. reported that patients with different *ACTG1* mutations in DFNA20/26 showed little difference in the age at onset and severity of HL^5^. However, this observation was based on only 2 families. Our data set was larger than that of the previous report and we were able to identify more accurately the phenotypes of *ACTG1-*associated HL. Figure 2A shows the results of a regression analysis of the hearing deterioration for all *ACTG1-*associated HL patients identified. These results indicate that hearing levels equivalent to 30dBHL or more were observed at about 14 years of age. The estimated onset age for *ACTG1*-associated hearing loss is, therefore, considered to be about 14 years old. The audiograms of two patients with *ACTG1* mutations (Family 3 III-5, and Family 12 IV-2 (Fig. 1)) appeared to be normal. Therefore, we regarded these non-symptomatic carriers to be too young (9 y.o. and 11 y.o.) to present with HL phenotypes.

Non-muscle cells in vertebrates, including cochlear hair cells, contain two different actin isoforms (β- and γ-cytoplasmic actin)^11^. In the mammalian cochlea (especially in the outer pillar cells, inner pillar cells and hair cells), γ-actin is more highly expressed than in the other cells^25^. The function of γ-actin is primarily to reinforce and repair the actin cytoskeleton and actin filaments, which are essential for the shape and function of the hair cell stereocilia^26^. Alterations in actin filament regulation caused by a mutation in actin-binding proteins was proposed as an important mechanism underlying *ACTG1-*associated HL^27^. Morin *et al*. showed that the post-lingual and progressive character of the hearing loss associated with *ACTG1* mutations can be the result of a progressive deterioration of the cytoskeletal structures of hair cells over time, based on *in vivo* experiments and *in vitro* biochemical analysis using NIH/3T3 cells^5^. We also used the NIH/3T3 cell line to assess the ability of the newly identified γ-actin mutants to be incorporated into the cytoskeleton based on the report of Morin *et al*. As a result, we found that the mutant γ-actins with p.I34M p.M82I, p.K118M and p.I165V mutations formed aggregates, whereas WT and p.R37H, p.G48R, p.E241K and p.H275Y mutations were distributed throughout the entire cell body and co-localized in the cytoskeleton based on phalloidin staining.

Thus, we speculated that some of the mutant γ-actins were not incorporated into the actin network required for stereocilia formation, resulting in HL. However, some other mutant actins localized in the actin cytoskeleton in a manner similar to that of the WT. Possible explanations for this discrepancy include 1) the pathogenicity classification of these variants with a normal γ-actin distribution pattern was incorrect and these variants were neutral or benign, and 2) the pathological mechanisms of these mutants were different from those of other mutations. Although these mutant γ-actins were incorporated into the actin network and showed normal distribution patterns, the function required for hearing might still have been disrupted.

In conclusion, we identified 13 *ACTG1* variants from 15 families. The rate of deterioration of HL was 1.7 dB/year and high-frequency HL progressed more rapidly than did that in the lower frequencies. This is the largest population of *ACTG1-*associated HL cases reported and is important for a better understanding of *ACTG1-*associated HL. From the results of our genetic analysis, clinical features as well as family segregation analysis, we regard the identified variants to be the genetic cause of HL in these patients. However, it is impossible to confirm that these variants were truly the cause of HL or not and further functional analyses are required.

## Supplementary information

Supplementary Information.
